# FFO-based controller for 3-phase inverter to reduce power quality problems in PV-integrated microgrid system

**DOI:** 10.1371/journal.pone.0336789

**Published:** 2025-12-10

**Authors:** Narisetti Ashok Kumar, M. Kiran Kumar, B. Srikanth Goud, Joon-Ho Choi, Ch. Rami Reddy

**Affiliations:** 1 Department of Electrical and Electronics Engineering, Koneru Lakshmaiah Education Foundation, Guntur, India; 2 Department of Electrical and Electronics Engineering, School of Engineering, Anurag University, Hyderabad, India; 3 Department of Electrical Engineering, Chonnam National University, Gwangju, Korea; 4 Department of Electrical and Electronics Engineering, Vallurupalli Nageswara Rao Vignana Jyothi Institute of Engineering and Technology, Hyderabad, India; 5 Applied Science Research Center, Applied Science Private University, Amman, Jordan; Wuhan University, CHINA

## Abstract

Renewable Energy Sources (RESs) are extensively utilized in the energy sector to meet the present energy demand. As a result of the excessive use of allotted resources, converters must be used numerous times to synchronize the power grid, resulting in low-quality power. The uncertainties resulting from the integration of multiple energy sources were reflected by the distribution system. As microgrids (MGs) transition, the main Power Quality (PQ) issues like voltage unbalancing, voltage swell/sag, poor power factor, power transients appear and Total Harmonic Distortion (THD). Numerous researches studies were going on for reducing PQ issues as well as improving the system reliability under all circumstances, but those models have some impact for attaining a good power flow at the end users. In this study, a microgrid including PVs, wind turbines, and batteries was constructed as a Distributed Energy Resource (DER). To address the aforementioned PQ difficulties, a unique regulating system has been proposed to manage the power flows. The input of the proposed optimal controller was considered as dc voltage, coupling voltage and load current, based on these values, the controller generated a pulse signal of a three-phase inverter to decrease the power supply from PV and wind to maintain a constant frequency and power factor. The optimal problem of the proposed controller was solved through the use of Fennec Fox Optimization (FFO). The performance of the FFO-based controller was analyzed under various PQ issue conditions. The suggested controller’s functionality was verified by expanding the microgrid to create a large, three-phase structure. The realistic microgrid’s feasibility is verified by the inclusion of demand response, line impedance, and off-nominal scenarios. The proposed model offers 2.2% THD, 50 Hz, 0.8 power factor at a simple microgrid. The proposed model provides well-mitigated performance in any circumstance with a constant frequency and power factor.

## 1. Introduction

The development of renewable energy sources, especially solar and wind energy, can meet the need for energy. However, connecting the sources of renewable energy to the utility grid results in problems with the quality of the power [1–2]. The PQ issues are sag, swell, voltage fluctuation and harmonic distortion. In order to prevent output power fluctuations, an adequate control system is required because solar irradiance and wind speed are dependent on atmospheric conditions [3]. In order to meet future energy demands, the AC-DC mixed hybrid microgrid has now been introduced [4–6]. A model-predictive approach is also used to control the boost converter and inverter [7–10]. Reducing the quality function is the major goal of predictive-based control. To balance grid operator demand, net DC voltage regulation is a quality function [11–12]. By modulating the inverter circuit, a predictive control method could also be employed to run the wind turbine-based permanent magnet synchronous generator [13–15].

A four-switch inverter has the benefit of having few switching losses, but cannot be employed at the megawatt level, which was developed to reduce the cost of inverters [16–18]. The output of the inverter is controlled using a variety of control methods, such as Proportional-Integral (PI), fuzzy, and predictive control [19–20]. The model predictive control system, which is also used to run the MW system, defines the inverter-firing and rectifier angles as control inputs [21–23]. The finite control sets prediction technique can be used by a hybrid energy system to regulate a 3-phase quasi-Z-source inverter with an unstable load and lessen ripples in the inverter current.

The goal of any control is to improve power quality by reducing output voltage and current variance [9]. However, the primary grid is being supplemented with renewable energy sources using the grid-connected inverter. Despite having a sophisticated construction, model predictive control is one of the most straightforward ways to use these inverters [10]. In order to minimize errors between computed values and anticipated values, the model predictive controller selects the precise switching state of an inverter [11]. The converter’s output is regulated by the maximum voltage vectors that reduce the cost [12]. To more effectively manage the grid-connected wind energy-based system, a fuzzy control technique and quasi-Z source type three-phase inverters were developed recently [13]. Those models have some drawbacks, such as lower reliability, higher cost, lower performance, and complex design. An innovative optimization-based controller for a three-phase inverter is needed to enhance PQ in an integrated microgrid system. A grid-connected hybrid system’s power quality is a major concern in the proposed work. This proposed model is effective to operate, high reliability and improves power quality.

- On the source side, the grid is designed to supply the power to the load. During PQ issues, PV and wind are utilized for load demand.
- A three-phase inverter is linked into the system for the power converter. The pulse generation of the three-phase inverter is accompanied by the use of a FFO algorithm-based controller.
- The pulse generation of the inverter is based on reference reactive and active power, 3-phase voltage and current, and the switching frequency of the system. These parameters are fed to the proposed controller to generate a suitable PWM signal for the inverter.
- The effectiveness of the suggested controller has been tested for line impedance in both on-grid and off-grid scenarios, and the result is for a realistic microgrid topology with PQ issues.

The remaining part of the manuscript is structured as follows: Section 2 states that some of the present works are reviewed, which are related to improving PQ in a system. Section 3 contains the overall design and process of the proposed work. The performance validation of the proposed model is presented in Section 4. The overall conclusion of the work is provided in Section 5.

## 2. Related work

Improvement of PQ in a system is considered an important task for a power system. Numerous models were introduced to mitigate PQ issues, among them, a few approaches which was recently developed models were reviewed as follows.

Ashok Kumar and Indragandhi [24] suggested that the main objective is to assist the accurate and structured containment of wind energy systems to the grid using a static compensator in conjunction with a battery energy storage system. This system offers a STATCOM- BESS-integrated controller for a grid-linked wind system. However, the functioning of this approach with a unity power factor is not appropriate. Goud and Rao [25] implemented the proportional-resonant controller-based synchronization techniques. A smart way to lessen the PQ difficulties is to develop a dual second-order generalized integrator PLL-based proportional resonant controller for a hybrid system. The biggest shortcoming is that the combination may intensify existing PQ problems in a system, namely, harmonic interruptions, sags and swells. Kumar [26] presented a method for enhancing the quality of the power coming from a grid-connected solar power system by employing a distribution static compensator. Although this system employs two-step control mechanisms, it is complicated.

Ren et al. [27] described a model to enhance the PQ being transferred in a solar PV-WE hybrid system, which is associated with a grid. Both the AC loads and the hybrid system’s power output are constantly disturbed. Reactive power imbalance results from this, which also increases voltage instability and power quality problems. Rao et al. [28] presented to improve the power quality characteristics at the three-phase distribution system’s point of common connection, an adaptation-based control strategy is devised. The control technique was intended to balance three-phase currents and take into account the system’s reactive power, but the cost is substantial. Garcia-Torres et al. [29] presented to ensure that the requirements are met, model predictive control is used to manage the microgrid power converters. The control algorithm is designed to work with the microgrid regardless of whether it is connected to the utility grid, operating independently, or connected to other microgrids. However, the model cannot provide a stable output during the fault period.

Sindi et al. [30] presented a distributed power condition controller, an improved custom power device, and a multi-microgrid system with interconnected modes and high penetration of various distributed generators on the island might all enhance the power quality, but the costs would be prohibitive. Salem et al. [31] developed a standalone DG inverter’s selective harmonic compensator and proportional resonant controller. In addition, the triple-action controller design is used. However, it is not a sag and swell refuse. Das et al. [32] suggested a method utilizing shunt hybrid filters. The presentation of SHF was examined by a regulating method known as Adaptive Fuzzy-Neural Network Control in order to produce an effective SG functioning in various situations of supply and load voltages. Despite the controller’s excellent performance, the system is unstable.

Morey et al. [33] suggested a Z-source DC–DC converter architecture, which has high gain and increased conversion efficiency for low-voltage PV applications. The proposed DC–DC converter is connected at the first stage, which provides very high gain at low-value duty cycle and offers very good efficiency. An ANFIS and PSO-based MPPT algorithm offers an extremely fast dynamic response with high accuracy and low reduced steady-state oscillations.

Gali et al. [34] developed an ADPM-based EISCT that not only utilizes a single grid side converter (GSC) but also reduces the sensor requirement and control structure complexity and thereby eliminates communication mismatch. The first segment focuses on active power management control through ADPM to facilitate efficient power flow between the utility grid and microgrid sources, which helps in ensuring efficient power sharing. The second component is dedicated to synchronizing the renewable energy sources of the microgrid with the utility grid and simultaneously maintaining PQ compliance as per IEEE 519–2022 standards. Ahmadi et al. [35] introduced a design, control, and power management scheme of a solar, wind and battery energy storage system (BESS) based microgrid (MG) system. The grid synchronization of the system is achieved by employing the power balance (PB) theory-based control algorithm. This microgrid system is managed and controlled by an improved power management (IPM) algorithm to enhance the power quality, stability, and efficiency. The literature from previous research papers is summarized in the Table 1, as follows.

**Table 1:** Summary of the literature.

Author name	Methods	Advantage	Limitations
Goud and Rao [25]	GWO, UPQC, FOPID	FOPID controller with GWO-based control techniques is implemented in the UPQC device, which operates the HRES to compensate for the PQ problems and meet the required load demand.	The combination of controllers may intensify existing PQ problems
Kumar and Kumar [26]	DSTATCOM, FLC, ADALINE-LMS	Two-stage controllers are utilized in this system, i.e., a step-up DC-DC converter in the first stage and a voltage source DC-AC boost converter in the second stage.	It is complicated to provide an output.
Garcia-Torres et al. [29]	MPC, FCS	The final power quality obtained in the microgrid depends on the exchanged power flow between its local devices connected and the grid	Cannot provide a stable output during the fault period
Sindi et al. [30]	APQC, TCSC, SAPF, swarm intelligence-based puzzle optimization, PID	TCSC and SAPF give the APQC scheme more reliability, robustness, and low cost. The APQC is controlled by two separate PID controllers for series and shunt devices.	The costs would be prohibitive
Das et al. [32]	AFNN, SAHF	The proposed filter can compensate the harmonic disturbances faster and with a lower percentage of THD	The system is unstable
Peter et.al., [43]	COA – Optimized FOPID controller	The COA-based FOPID controller works on a wide range of microgrid configurations with various operating conditions and dynamics	Load dynamics are not verified
Upadhaya et.al., [44]	GZA-based FOPID controller	With this controller, the fuel cost is minimized. The proposed controller deregulated the frequency at various DER systems.	System stability still needs to be improved.
Hussain et.al., [45]	(1 + FOPID) controller	The wild horse optimizer-based FOPID controller regulates the frequency of the interconnected system to stabilize.	Not verified for the variations in the load dynamics.

The aforementioned literature focuses on problems with PQ in a microgrid which is run in either an independent mode or a mode that is connected to the grid, and all of these techniques have limitations like not mitigate PQ issues [21], not stable operation [19,22], high cost [18], complexity [16] and poor power factor [14]. Furthermore, the majority of these methods do not take into consideration all the typical aspects of the power quality problem. Regulations governing the production and use of energy are always changing, necessitating a constant search for novel approaches that work.

## 3. Proposed methodology

**Fig 1:**
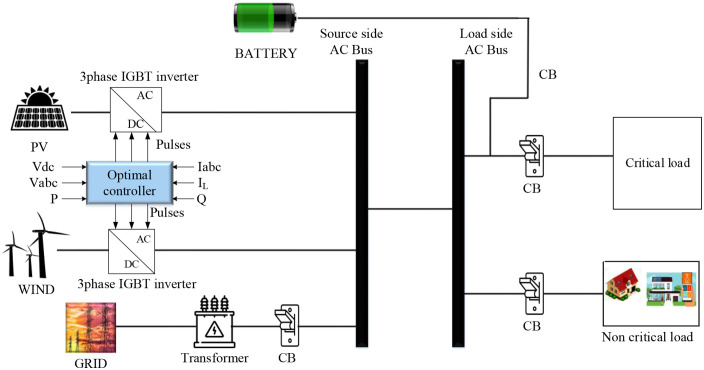
Schematic model of proposed PQ mitigation model.

Voltage and current quality together make up power quality. Electrical systems are designed to function under sinusoidal voltage waves with consistent magnitude and frequency. Any deviation from this predetermined magnitude and frequency can be regarded as a PQ issue. Numerous studies concentrate on increasing power quality. However, there are certain drawbacks. In order to overcome the flaw in the existing model, a novel optimal algorithm-based controller was proposed. The proposed optimal controller analyzes voltage, current, load current, and real and reactive power to generate a pulse signal of a converter. Fig 1 below clearly depicts the suggested model’s architecture.

A standard microgrid system with loads is included in the proposed architecture. Renewable energy sources, namely wind, solar, batteries, and the utility grid, are regarded as the source side. To convert the source electricity, place a DC to AC converter on the source side. Loads are split into two types such as critical and non-critical loads. Critical loads are always energized using a battery source, whereas MG powers the non-critical loads. Wind energy, PV array, BESS, line impedance, inverter, loads, and utility grid make up the MG organization. The CB of the grid source is utilized for the ON/OFF process of grid power, same as the non-critical load CB is also utilized for the ON/OFF process. The critical side CB is utilized for ON/OFF the sources like PV/wind or grid source during the poor battery supply period, to avoid fluctuation in load supply. Power quality issues arise in the system, which cause frequency, voltage and power factor fluctuation. To overcome these, an optimal controller is placed on the source side to connect PV and wind. Nine-element inputs, including three-phase voltage and current, load current and reference reactive and real power, are considered. Based on these parameters, the controller generates six pulses for the three-phase inverter. Based on the gate pulse, the system model effectively overcomes PQ issues. Below is a list of the proposed model’s mathematical model [34–36].

### 3.1 PV panel model

**Fig 2:**
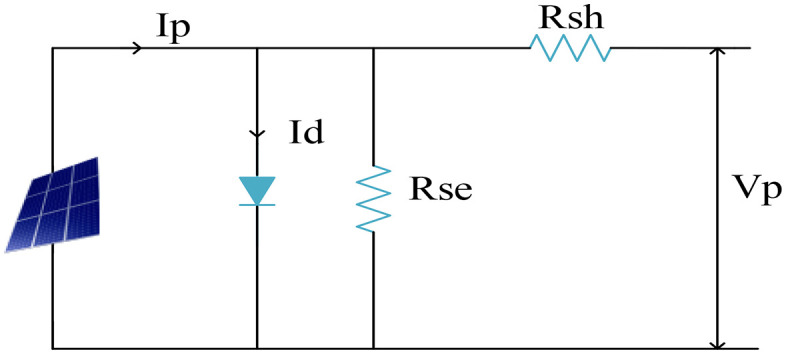
Equivalent circuit of PV.

Numerous solar cells are linked in parallel and series to form a solar PV array [7]. Many solar cells must be joined together to generate the required amount of power. Fig 2 displays the PV model’s corresponding circuit. In that $I_{p}$ denotes photon current, $R_{sh}$ signifies shunt resistance, $R_{se}$ represents series resistance and $V_{p}$ denotes output voltage.

The physical representation of solar radiation on an inclined surface is provided by,


$$I_{T}=I_{b}R_{b}+I_{d}R_{d}+\left(I_{b}+I_{d}\right)R_{r}\;$$ (1)

Where, $I_{b}$ signifies normal solar irradiance and $\;I_{d}$ signifies diffused solar irradiance. $R_{d}$ and $R_{r}$ are the diffusion part of the solar irradiance and tilt parameter of reflected, respectively. The models’ hourly PV output is stated as,


$$P_{sj}=\eta_{r}\eta_{pc}\gamma A_{PV}I_{TJ}\left(\text{1}-K\left(\text{1}-K\left(\left(T_{a}+\frac{I_{T,\;NOCT}}{\left(NOCT-T_{a,\;NOCT}\right)}I_{T}\right)-T_{r}\right)\right)\right)\;\;\;\;$$ (2)

where, $\eta_{r}$ signify the module’s reference efficiency,$\;\eta_{pc}$ suggests the efficiency of smoothing and conditioning power, $A_{PV}$ denotes a photovoltaic area, γ signify the cell’s density factor in the module, K indicates the temperature coefficient, $T_{a}$ indicates instantaneous ambient temperature, $T_{r}$ denote reference temperature, $\;I_{T}$ signify monthly temperature, and NOCT indicates cell temperature in normal operating. PV (photovoltaic) systems are grid-connected using an interface converter that uses maximum power point tracking to feed the grid with sufficient solar energy as is allowed.

### 3.2. Wind turbine model

Wind turbines first transform the kinetic energy associated with the wind’s dynamic motion into rotational energy, which is then matched to the speed of the generator and turbine by the movement of the gearbox [25]. The generator converts the mechanical energy of the turbine into electrical energy. The windmill can produce 1 kW of power.


$$P_{m}=\frac{\text{1}}{\text{2}}\rho c_{p}A_{r}V_{W}^{\text{3}}$$ (3)

Where ρ denotes air density in $\;\frac{g}{m^{\text{3}}}$,$\;P_{m}$ is the mechanical power generated from wind in watts, $\;A_{r}$ is the area of the turbine rotor in $\;m^{\text{2}}$, $\;c_{p}$ is the power coefficient, $\;V_{W}$ represents the speed of the wind in $\frac{m}{s}$.

### 3.3. Battery modelling

A battery energy storage system (BESS) is a type of device used to store electric power by using batteries. It can store electricity under ideal conditions and can supply electric power under situations where there is a shortage of power [5]. The most common type of BESS utilized for PV arrays to produce the biggest battery capacity is lead-acid batteries. The battery is programmed to charge and discharge at 80% and 20% of its full charge, respectively [8]. The formulae for battery charge and discharge voltage are provided as,


$$V_{bat}=V_{\text{0}}-Ri-K\frac{Q}{Q-it}\left(it+i^{*}\right)+\text{exp}(t)$$ (4)

$$V_{bat}=V_{\text{0}}-Ri-\left[K\frac{Q}{it-\text{0}.\text{1}Q}\right]i^{*}-\left[K\frac{Q}{Q-it}\right]\text{it}+\text{exp}(t)$$ (5)

Where $V_{\text{0}}$ is battery constant voltage(V), $V_{bat}$ is battery voltage, Q is the battery charge $\left(\frac{V}{Ah}\right)$, K is polarisation constant (V), $i^{*}$ is filtered current (A), it=∫i dt signifies actual battery charge(Ah), R denote internal resistance (Ω), and i represent battery current (A).

### 3.4. Utility grid modelling

The device is linked between various voltage levels using transmission lines and complete transformers. The grid system is created using a Thevenin equivalent circuit gathered through a series of sine wave voltages of (Z=R+jX) impedance at a frequency of (220 V, 50 Hz).


$$P_{utility}=\frac{\Delta V}{I}$$ (6)

Where I represents current, and ΔV denotes potential difference.

### 3.5. Load

The load is the object that consumes electrical energy. In other words, an electrical load is equipment that, after absorbing electrical energy through the use of current, transforms electrical energy into other forms, such as light, heat, work, etc. Critical and non-critical loads are two types of loads.

i ***Critical load***

Critical loads are those that have a direct impact on an organization’s ability to function and that must either continue to operate (without a power pause) when their mains supply fails or be turned off gradually to avoid system failures, data corruption, and life-shortening effects. Example: Hospital, Industry, Laboratory

ii ***Non-critical load***

In order to preserve additional battery uptime for the critical Load to safely shut down, additional equipment, such as monitors and other peripherals, should be attached to non-critical load outlets (NCL outlets). Example: Domestic appliance

### 3.6. Three-phase IGBT inverter model

A three-phase bridge inverter with six switching devices coupled in a bridge alignment may be used to symbolize a three-phase inverter. The forced commutated insulated gate bipolar transistor (IGBT) diode is used to represent the three-phase bridge converter. When the forward voltage is set to 0, the IGBT-diode’s internal resistance $\left(R_{on}\right)$ is 1mΩ. A three-phase PLL, a voltage direct current (*V*_*DC*_) controller, a current regulator, and a PWM unit make up the four primary components used in the proposed optimal controller. The PLL unit needs to be provided with line-to-line voltage $V_{abc}$and line current$I_{abc}$ in order to synchronize and measure current and voltage. The PLL transforms the reference signal abc components and ωt angle of phase into the appropriate dq parameters. The solar panel DC voltage output $V_{dc}$is matched with a reference voltage $V_{dcref}$ by the VDC controller, which uses a PI regulator to limit the real current component $I_{dref}$as a reference signal. The PLL and VDC regulator blocks’ output signals are fed into the current regulator block, which uses an internal PI controller to produce the and $V_{dconv}$ and $V_{qconv}$voltages. If $I_{d}$ denotes positive in the inverter operating mode, this converter generates active power, and if $I_{q}$ denotes positive in the inductive mode, it generates reactive power. The measured variables $V_{dconv}$,$\;V_{qconv}$ and ωt from PLL units and current regulator, respectively, are converted back to reference component$U_{abc-ref}$. A gripping and delaying unit are denoted as $U_{abc-ref}$. Before this signal is given to the PWM unit, the designated sampling period during the model’s simulation is clearly crossed. The PWM generator generates the IGBT firing pulses by crossing a delay unit once more, and the reference voltage $U_{ref}$.

**Fig 3:**
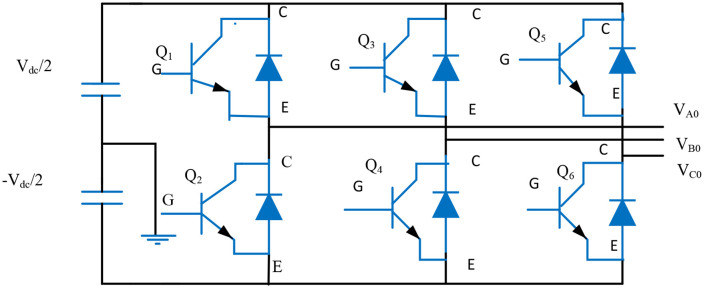
Three-phase Inverter model configuration.

Fig 3 illustrates this situation, where $V_{A\text{0}}\sim V_{C\text{0}}\;$ represents the voltage of the three-phase power network and $i_{a}\sim i_{c}$ represents the positive direction of the parallel grid’s current.

When the grid voltage is three phase symmetry, the inverter bridge’s output voltage, $v_{a}\sim v_{c}$ ignores the high-frequency component and the three-phase circuit is independent of one another [25]. The three phase grid-connected inverter mathematical model in the ABC coordinate system is displayed in (7).


$$\left[\begin{array}{c} L\frac{di_{a}}{dx}\\ L\frac{di_{b}}{dx}\\ L\frac{di_{c}}{dx} \end{array}]=[\begin{array}{ccc} {-R}_{s} & \text{0} & \text{0}\\ \text{0} & {-R}_{s} & \text{0}\\ \text{0} & \text{0} & {-R}_{s} \end{array}][\begin{array}{c} i_{a}\\ i_{b}\\ i_{c} \end{array}]-[\begin{array}{ccc} \text{1} & \text{0} & \text{0}\\ \text{0} & \text{1} & \text{0}\\ \text{0} & \text{0} & \text{1} \end{array}][\begin{array}{c} v_{a}\\ v_{b}\\ v_{c} \end{array}]+[\begin{array}{ccc} \text{1} & \text{0} & \text{0}\\ \text{0} & \text{1} & \text{0}\\ \text{0} & \text{0} & \text{1} \end{array}][\begin{array}{c} V_{A\text{0}}\\ V_{B\text{0}}\\ V_{C\text{0}} \end{array}\right]$$ (7)

Physical quantities in static coordinates can be turned into physical quantities in the two-phase synchronous rotating dq coordinate system using coordinate transformation, as shown in eqn. (8).


$$\left[\begin{array}{c} L\frac{di_{d}}{dx}\\ L\frac{di_{q}}{dx} \end{array}]=[\begin{array}{cc} {-R}_{s} & \omega t\\ \omega t & {-R}_{s} \end{array}][\begin{array}{c} \;i_{d}\\ \;i_{q} \end{array}]-[\begin{array}{cc} \text{1} & \text{0}\\ \text{0} & \text{1} \end{array}][\begin{array}{c} \;v_{dconv}\\ \;v_{qconv} \end{array}]+[\begin{array}{cc} \text{1} & \text{0}\\ \text{0} & \text{1} \end{array}][\begin{array}{c} \;V_{d}\\ \;V_{q} \end{array}\right]$$ (8)

a *Controller design of proposed optimal PWM generation*

**Fig 4:**
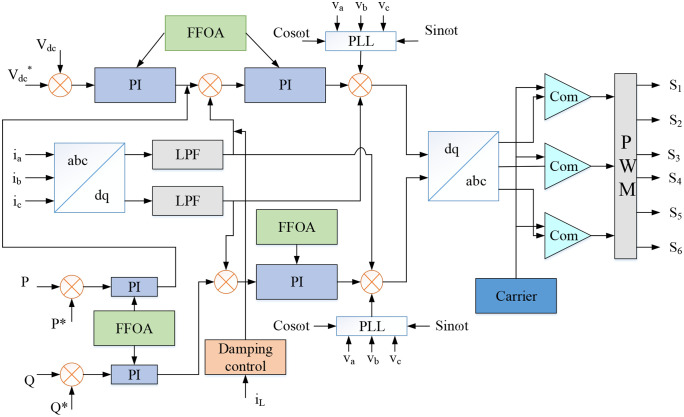
Controller design of proposed optimal PWM generation.

By utilizing the controllers, the dynamics (stability) and quality of the power given to the system are improved, leading to the system’s good performance. The proposed optimal controller is illustrated in Fig 4. The input of the optimal controller is considered as real power (P), reactive power (Q), three-phase voltage $\left(V_{abc}\right)$ and current $\left(I_{abc}\right)$ and load current $\left(I_{L}\right)$. Those inputs are compared to its corresponding references to make an error signal. Based on these error signal to generate a pulse signal.

In a hybrid distribution system, the simultaneous effects of PQ parameters, namely THD, voltage deviation, power factor and frequency, have been evaluated and controlled under various conditions. It is planned to have a standard microgrid with a load system made up of the utility grid, source side PV, and batteries. A three-phase inverter has been linked to the system power converter. To make sure the inverter generates the best possible pulses, a controller based on the Fennec fox optimization method is utilized. The inverter pulse generation depends on the system’s switching frequency, reference active and reactive power, 3-phase voltage and current, and other factors. These parameters are given to the proposed controller in order to produce an appropriate PWM signal for the inverter. The efficiency of the suggested controller and the impact of line impedance on losses and power sharing in both off-grid and on-grid scenarios have been researched for actual microgrid designs. To handle the system’s stability and dependability in both grid-unconnected modes and grid-connected mode, the best controller is proposed. The parameter of the proposed controller is optimally selected by using the FFO algorithm. The proposed control organization is divided into four sets for optimization. The following types of control structures,

i *Voltage control*

After measuring the inverter’s voltages, the axis is changed to direct and quadrature. Based on the result that was compared to the reference value, the error value was calculated. The controller receives the error value and any changes to the error value. The error value is reduced by using the PI controller with FFOA.

ii *Current control*

The three-phase signals are synchronized with a PCC voltage through phase locked loop (PLL). To ascertain the dc component of id and iq, a dq current component is sent through the low pass filter. The reference current and real current are computed for error calculation in current control. The controller’s inputs include the error current and error current change. The controller must adjust the gain parameters for the current to flow more efficiently.

iii *Reactive power control*

The control mechanism is put into action by the planned controller. The reactive power, both reference and actual, is determined for error computation. Reduced error values are made possible by the controller. Initial computations are done on the three-phase currents, and then they are translated to quadrature and direct axis. The controller reduces the error numbers.

iv *Damping control*

The line current magnitude provides a control pulse to this controller. The damping controller is run using the transfer function as a basis. The washout stage and the first order lead leg compensator block are both parts of the transfer function. The microgrid is connected to the damping control approach to control line current magnitude [25].


$$F(d)=G.\frac{sT_{w}}{\text{1}+sT_{w}}.\left[\frac{\text{1}+sT_{\text{1}}}{\text{1}+sT_{\text{2}}}\right]$$ (9)

Where G denote the transfer function, T denote the time constant and s denotes the complex frequency controller parameter.

v *Droop control*

The droop control process alters the output of reactive power (Q) and active (P) to replicate the droop features of the power system. Through altering the output, the output voltage’s frequency and amplitude are managed. The droop control mechanism regulates reactive and real power. The starting point for reactive and actual power of inverter currents and voltage in the MG structure [25]. Eqn. (10) shows the inverter’s three-phase output voltage:


$$\left[\begin{array}{c} V_{a\text{1}}\\ V_{b\text{1}}\\ V_{c\text{1}} \end{array}]=[\begin{array}{c} V_{m}\text{cos}\left(\omega t\right)\\ V_{m}\text{cos}\left(\omega t-\frac{\text{2}\pi }{\text{3}}\right)\\ V_{m}\text{cos}\left(\omega t+\frac{\text{2}\pi }{\text{3}}\right) \end{array}\right]$$ (10)

The symbols in eqn. (11) denote the inverter’s three-phase current:


$$\left[\begin{array}{c} I_{a\text{1}}\\ I_{b\text{1}}\\ I_{c\text{1}} \end{array}]=[\begin{array}{c} I_{m}\text{cos}\left(\omega t\right)\\ I_{m}\text{cos}\left(\omega t-\frac{\text{2}\pi }{\text{3}}\right)\\ I_{m}\text{cos}\left(\omega t+\frac{\text{2}\pi }{\text{3}}\right) \end{array}\right]$$ (11)

Where ω represents angular frequency, $V_{m}$ signify maximum voltage V and $I_{m}$ denote maximum current. Through the use of the park transformation, current and voltage readings from a three-phase inverter are converted into two axes of rotational coordinates. The output current and voltage of an inverter following Park’s transformation in Eqn. (12):


$$\left[\begin{array}{c} V_{\alpha }\\ V_{\beta } \end{array}]=V_{a\text{1}b\text{1}c\text{1}\to \alpha \beta }[\begin{array}{c} V_{a\text{1}}\\ V_{b\text{1}}\\ V_{c\text{1}} \end{array}]=[\begin{array}{c} V_{m}\\ \text{0} \end{array}\right]$$ (12)

Where $V_{\alpha }$ and $V_{\beta }\;$ are α−β coordinate arrangements. In the aforementioned transformation, the output current of inverter is given as,


$$i_{\alpha -ref}=\frac{P_{ref}}{V_{\alpha }}$$ (13)

$$i_{\beta -ref}=\frac{P_{ref}}{V_{\beta }}$$ (14)

It is possible to calculate power using the inverter output. Real and reactive power are computed during the power calculation. Using these actual and reactive power reference esteems, change in error and error value are computed. The values of $P_{ref}\;$and $Q_{ref}$ are associated with Q and P. Through the accompanying condition, the Q and P values are used to determine the voltage amplitude (E) and frequency (ω).

i The converter voltage angle that is shifting as ω, determines the active power (P) in large part.
ii Voltage amplitude E affects reactive power Q.

The reactive and actual power of droop control in SG structures is enabled by these two conditions. Below are the results of the calculations for voltage amplitude and frequency.


$$\omega =\omega^{*}-S_{\alpha }(s).\left(p-p^{*}\right)$$ (15)

$$E=E^{*}-S_{\beta }(s).\left(q-q^{*}\right)$$ (16)

Where $\omega^{*}$ is nominal frequency, $S_{\alpha }(s)$ and $S_{\beta }(s)$ are constant droop coefficients, E denotes referred voltage amplitude, ω denotes referred frequency,$\;E^{*}$ denotes nominal voltage amplitude, q and p represent calculated reactive and real power,$\;\;p^{*}$ and $q^{*}$ signify the reference value of reactive and real power. The following equation is used to determine these proportional constants.


$$S_{\alpha }(s)=\frac{\Delta \omega_{max}}{P_{max}}=r$$ (17)

$$S_{\alpha }(s)=\frac{\Delta E_{max}}{Q_{max}}=s\;$$ (18)

Where s denotes the reactive power coefficient, r denotes the real power coefficient,$\;\Delta \omega_{max}$ signifies the maximum allowed voltage amplitude droop,$\;\Delta E_{max}$ signifies the maximum acceptable frequency,$\;Q_{max}$ signifies the maximum allowable reactive power and $P_{max}$ signifies the maximum acceptable real power. The voltage formation is then completed using these two numbers for frequency and voltage amplitude. In order to manage voltage and current, the three-phase voltage is transformed to α−β axis via the Parks transformation after the $V_{ref}$ voltage is calculated through voltage formation. At that moment, the proposed controller is put into action, controlling voltage and current. Once more, a three-phase output voltage is created from the output of the current and voltage control. Voltage is finally sent to the inverter using PWM. The actual and reactive power are regulated by altering the frequency and voltage amplitude settings. This droop control technique overcomes the power management and PQ issues in this SG system. By using droop control, the load and generation sides of the system manage the power in the SG, increasing system efficiency and reducing power loss [37–38].

The droop controller is employed for power management in the SG, managing reactive power and actual power. The PI controller’s input is the error between the actual current and the reference current. The suggested controller aids in lowering error levels. The control structure has four PIs that are used to control the current and voltage in SG system. Controls the current and voltage in the voltage-current loop to match the reference current and voltage flowing across the inverter. The PI controllers must be correctly adjusted in order to produce the best outcomes possible from the control loops of the suggested technique. Here, the suggested algorithm FFO aids in achieving the right tuning procedure. The FIFO algorithm is utilized to tune voltage, real, current, and reactive power while minimizing error [39–42].

b *Background of FFOA*

The family of foxes, which includes the fennec fox, lives in North Africa’s dry Sahara Desert, Sinai Peninsula, and other regions. The smallest canid species, the fennec fox, can be recognized by its huge ears. The fur on the fennec fox is straw in colour. Its tapering tail has a black tip. Its enormous ears, which are covered in dense hair inside and have longitudinal reddish stripes on the back, conceal the external auditory meatus. On the back, the ear margins are darker but still pale. An omnivorous animal, the fennec fox eats fruits, certain tubers, skinks, mice, geckos, tiny birds, and their eggs. All other features are secondary to the two fennec fox behaviours. These traits include a potent digging method and a means of evading predators. More important than the others are the fennec fox’s two behavioural characteristics. Deep digging skills and the ability to run away from predators are a couple of these traits. Sand is where fennec foxes lay their nests. They quickly dig their meal out of the sand and consume the small animals and insects that they discover there because they are sensitive to movements underground. Pharaoh eagle-owls and other African horned owls consume the young of fennec foxes. Striped hyenas, jackals, and caracals may have been observed preying on fennec foxes, according to anecdotal reports. According to the nomads, the fennec fox is so quick and adept at shifting location that even their Salukis struggle to catch it. The fennec fox’s ability to dig for prey in the sand and its capacity to flee from an approaching predator are two examples of different types of intelligent techniques that the fennec fox uses as models for the proposed FFA optimization process.

c *Optimal parameter selection in PI controller*
i *Initialization*

Fennec foxes are search members in the population-based metaheuristic algorithm known as FFA. The positions of the fennec foxes in the search space in FFA each indicate a potential resolution to the problem and establish the values of the decision variables. Therefore, any fennec fox population is represented mathematically by a population matrix and expressed as a vector. In the proposed model, proportional gain and integral gain are initialized in the PI controller.


$$K_{p}=\left\{K_{p\text{1}},K_{p\text{2}},K_{p\text{3}},\ldots K_{pn}\right\}\;\;\;$$ (19)

$$K_{I}=\left\{K_{I\text{1}},K_{I\text{2}},K_{I\text{3}},\ldots K_{In}\right\}$$ (20)

Where $K_{p}\;$ proportional gain, $K_{I}$ is the integral gain, and n is the number of optimization.

ii *Fitness*

To get the best fitness function value, the problem restrictions during assessment must be met. Consequently, the definition of the overall fitness function is


$$\begin{array}{c} f=\frac{\text{1}}{n}\sum {\mathrm{\backslash nolimits}}_{i=\text{1}}^{n}{\left(Y_{i}-{\hat{Y}}_{i}\right)}^{\text{2}} \end{array}$$ (21)

Where f is the fitness function, n is the number of data points,$\;Y_{i}$ is the observed value,$\;\hat{Y}$ is the predicted value.

iii *Updation*

The value of the objective function serves as the standard by which to judge which of the potential solutions is the best one. The best candidate solution is chosen since it has the highest value for the objective function. Because candidate solutions are modified, the best candidate solution is also updated between iterations. FFA members’ current locations inside the search area have been updated using two common fennec fox actions. One of these actions is digging in the sand to eat prey, while another is escaping from a predator.

#### Digging to look a prey.

A neighbourhood with a radius denotes that the fennec fox’s real location is taken into consideration to replicate its behaviour while digging. The fennec fox can find a better answer if it does a local search in this area. Equation (22) to (24) is used to mathematically mimic this stage of updating FFA members.


$$x_{i,j}^{P\text{1}}=x_{i,j}+(\text{2}.r-\text{1}).R_{i,j}$$ (22)

$$R_{i,j}=\alpha .\left(\text{1}-\frac{t}{T}\right).x_{i,j}$$ (23)

$$X_{i}=\left\{ \begin{array}{c} X_{i}^{P\text{1}},F_{i}^{P\text{1}}<F_{i}\\ X_{i}\;,\;\;else \end{array},\right.\;$$ (24)

Where $x_{i,j}^{P\text{1}}$ is the j th  dimension, $X_{i}^{P\text{1}}$ is the new advised status of i th fennec fox based on the first phase, $\;\;F_{i}^{P\text{1}}$ is its objective function value, $\;R_{i,j}$ is the neighbourhood radius for $x_{i,j}$,  t is the iteration counter, T is the total number of iterations, and α is a constant set to 0.2.

#### Escape plan for a predator attack.

This fennec fox’s escape manoeuvre serves as the foundation for our mathematical model’s comprehensive search space scanning. The planned FFA’s exploring capabilities are improved through the simulation of this escape plan. It facilitates finding the best global solution by not getting bogged down in the best local ones. As a result, it is possible to use the random placement of each candidate solution within a search space as a fennec fox’s behaviour in its escape. The mathematical simulation of the second stage of the FFA population is updated by means of equations (25) to (27).


$$X_{i}^{rand}:x_{i,j}^{rand}=x_{k,j,}k\in \left\{\text{1},\text{2},\ldots ,\;N\right\},i=\text{1},\text{2},\ldots ,N,$$ (25)

$$x_{i,j}^{P\text{2}}=\left\{ \begin{array}{c} x_{i,j}+r.\left(x_{i,j}^{rand}-\text{I}.x_{i,j}\right),\;\;\;\;F_{i}^{rand}<F_{i},\\ x_{i,j}+r.\left(\text{1}.x_{i,j}-x_{i,j}^{rand}\right),\;\;\;\;\;\;\;\;\;\;\;\;else, \end{array}\right.\;\;\;$$ (26)

$$X_{i}=\left\{ \begin{array}{c} X_{i}^{P\text{2}},F_{i}^{P\text{2}}\\ X_{i},\;\;\;else \end{array}\right.\;$$ (27)

Where,$\;x_{i,j}^{rand}$ denote j th  aspect, $\;X_{i}^{rand}$ denote target position of i th fennec fox, $\;F_{i}^{rand}$ denote objective function value, $x_{i,j}^{P\text{2}}$ is the j th  dimension, $X_{i}^{P\text{2}}$ signifies the new suggested status of the i th fennec fox, I is a random number from the set, and $\;F_{i}^{P\text{2}}$ denotes objective function.

#### Termination.

**Fig 5:**
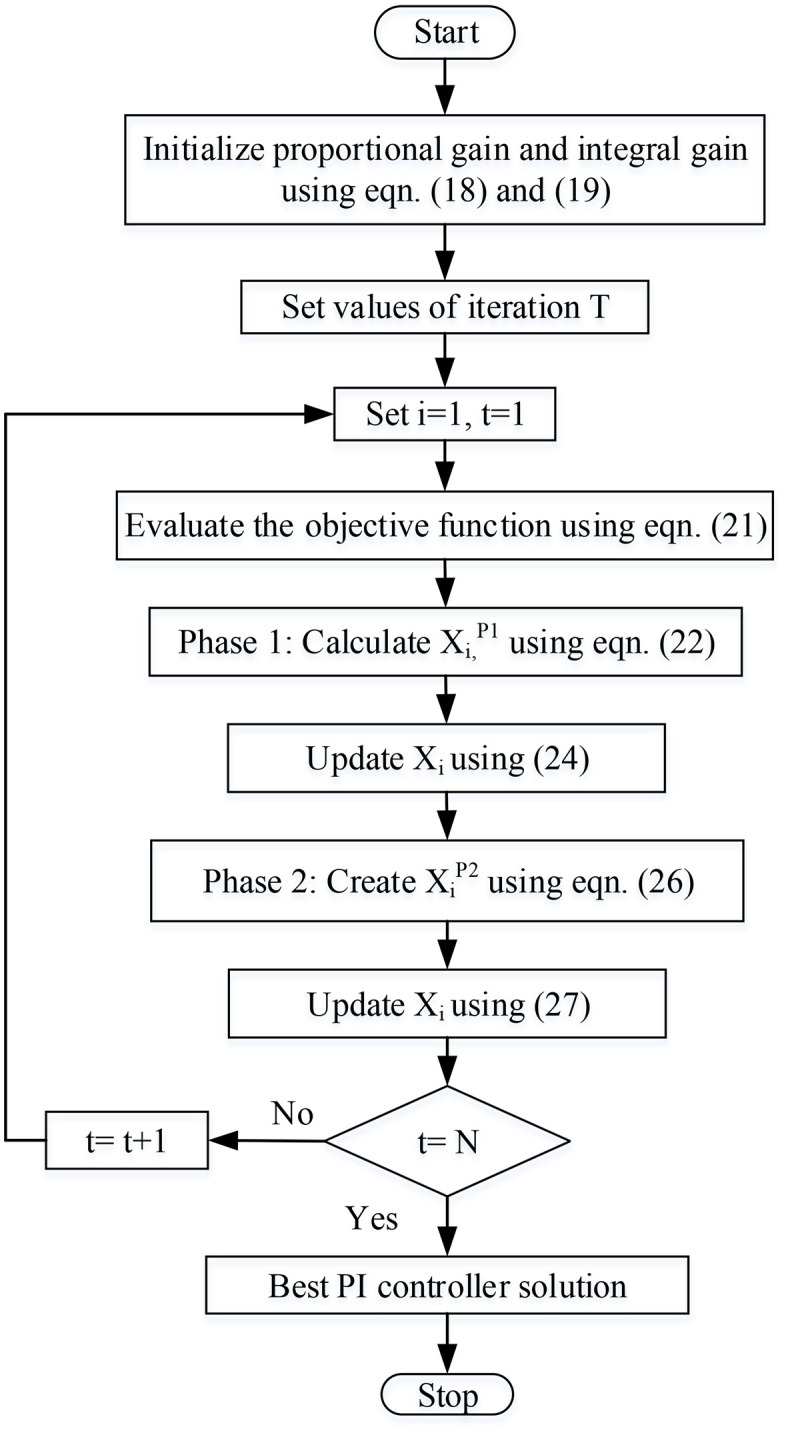
Flow chart of proposed model.

The process is repeated until the best value of the parameter is obtained. When the best solution is to get, the process goes to terminates. The proposed FFA optimization-based parameter selection flow chart is shown in Fig 5.

### 3.7. Pseudo-code of proposed FFA

Start FFA

Initialize proportional gain and integral gain using the equation. (19) and (20)

Set the number of iterations (T)

Generate an initial population matrix at random

Evaluation of the objective function using Eqn. (21)

*for n = 1: T*

*for f = 1: n*

Phase 1: digging in search of prey beneath the sand

Calculate $x_{i,j}^{P\text{1}}$ using (23)

Update using eqn. (24)

Phase 2: A plan for getting away from the predators’ attack.

Calculate $X_{i}^{P\text{2}}$ using (24)

Update using eqn. (27)

*end for*

Save the most effective remedy thus far.

*end for*

Output the best solution of PI parameter.

end FFA

The proposed controlling system provides a good performance at all periods, like sag, swell and interruption. Also, the controller maintains the frequency and power factor constantly. In proposed model is effectively done, and the performance analysis is given below.

## 4. Results and discussion

In the suggested model, the gate pulses to the inverter are controlled by the suggested optimal controller, enhancing the power quality. A three-phase AC microgrid at 10 kW is the suggested method for meeting the non-critical (6 kW) and critical (4 kW) demands. The critical load receives the majority of the demand fulfilment in case of grid supply failure or maintenance, especially if it is isolated. The combined active and reactive powers for the total load are defined as 7.5 kVar, 10 kW, and 400 V at a frequency of 50 Hz. The proposed model parameters and specifications are mentioned below. Table 2 shows the proposed model parameters and specifications.

**Table 2:** Proposed model parameter and specification.

Parameter	Specification
DC input voltage	800-900V
PCC voltage	400 V
PCC current	10A
PF at rated power	>0.98
Total capacity of microgrid	14 kw
Solar	4kw
Wind	4kw
Grid	6kw
Critical	4kw
Non critical	6kw
Battery	15Kwh
Frequency	50Hz

A small microgrid, a realistic microgrid and a microgrid with additional line impedances structure make up the suggested microgrid model, which is described in the Sub-sections. In the subsection below, the proposed control mechanism based on fennec fox optimization is described.

- Simple microgrid model
- Microgrid model with additional line impedances
- Realistic microgrid structure

The following three instances, which attest to the controller’s effectiveness, can be used to the suggested strategy.

### 4.1. Simple microgrid model

A small-scale microgrid has been planned, consisting of two DERs connected to the grid via PCC, with both non-critical and critical loads connected to the main load bus. In addition, a local BES system supports the essential load bus to ensure continuous supply in the event of grid or DER1/DER2 failure. It is acknowledged that given island conditions, two PVs (DERs) with the same rating introduced a significant quantity of harmonics. An MPPT model is made to monitor a PV system’s maximum power output. The converter topology causes them to produce higher voltage and current harmonics as well as PQ problems in the system when operating in an islanded mode. A 3-phase inverter that is operating properly to resolve these problems. A fennec fox optimization-based controller is proposed to generate the suitable pulse of the inverter. The 3-phase voltage and current, reference reactive and active power, load current, and switching frequency of a system are the foundations upon which the inverter generates pulses. The primary responsibility of the proposed controller is to keep THD below the permitted 5% range of IEEE Std. 519−2014. Additionally, PF is regarded as a significant factor in the PQ at the load demand. In order to prevent damage to equipment or devices from excessive current and heat flow, it is recommended that the PF be equal to or larger than 0.9. The allowable range of PF is stated in the appendix of the IEC 60831–1/2 standard. Performance analysis is done in a simple microgrid structure is explained in the section below.

**Fig 6:**
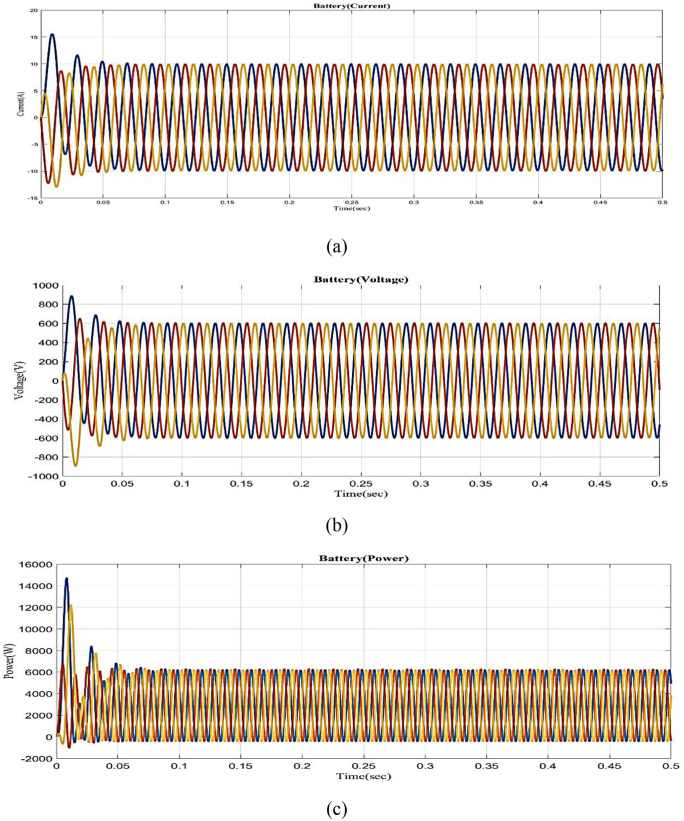
Analysis of Battery with bi-directional inverter (a) Current (b) Voltage (c) Power.

Fig 6(a) shows the horizontal axis in the graph denotes time, and the vertical axis is the battery with bi-directional inverter current. Here, lead acid batteries are considered, and a battery with a bi-directional inverter will fluctuate slightly in 0–0.04s and gradually reach 10A in 0.04–0.5s. The horizontal axis in Fig 6(b) depicts time, while the vertical axis reflects the battery with a bi-directional inverter. It fluctuates slightly between 0 and 0.03 seconds and consistently maintains a 600V voltage between 0.03 and 0.5 seconds. The battery with bi-directional inverter power vs time is shown in Fig 6(c) clearly. The power will fluctuate significantly between 0 and 0.06 seconds, but it will remain stable in 6000W between 0.06 and 0.5 seconds.

**Fig 7:**
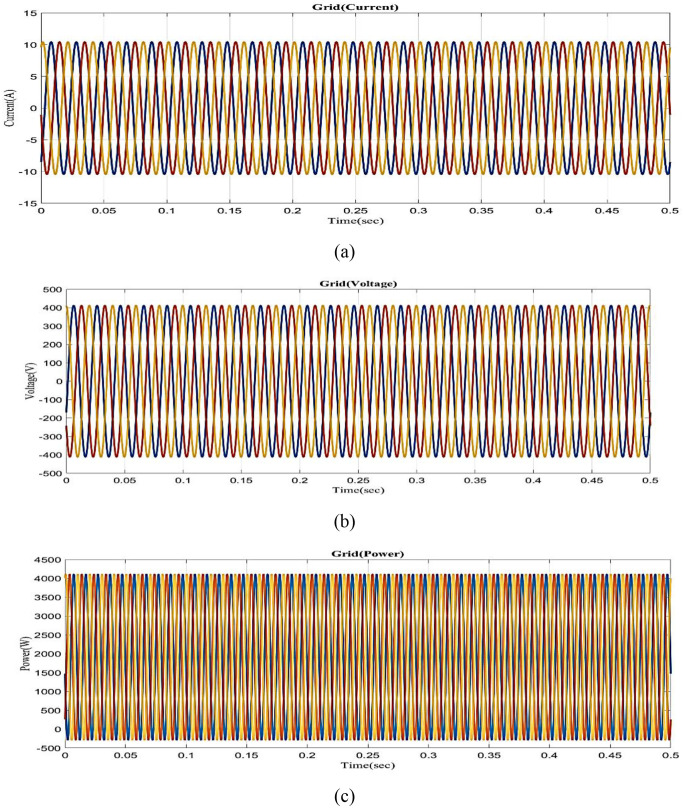
Analysis of (a) grid current vs time, (b) grid voltage vs time, (c) grid power vs time.

Fig 7(a) depicts the grid current versus the time graph. Grid current consistently maintains at 10A for 0 to 0.5 seconds. Fig 7(b) shows the grid voltage vs time graph. Grid voltage consistently maintains 400 volts in 0 to 0.5 seconds. Fig 7(c) shows the grid power vs time graph. Grid power is maintained at 4000W for 0 to 0.5s.

**Fig 8:**
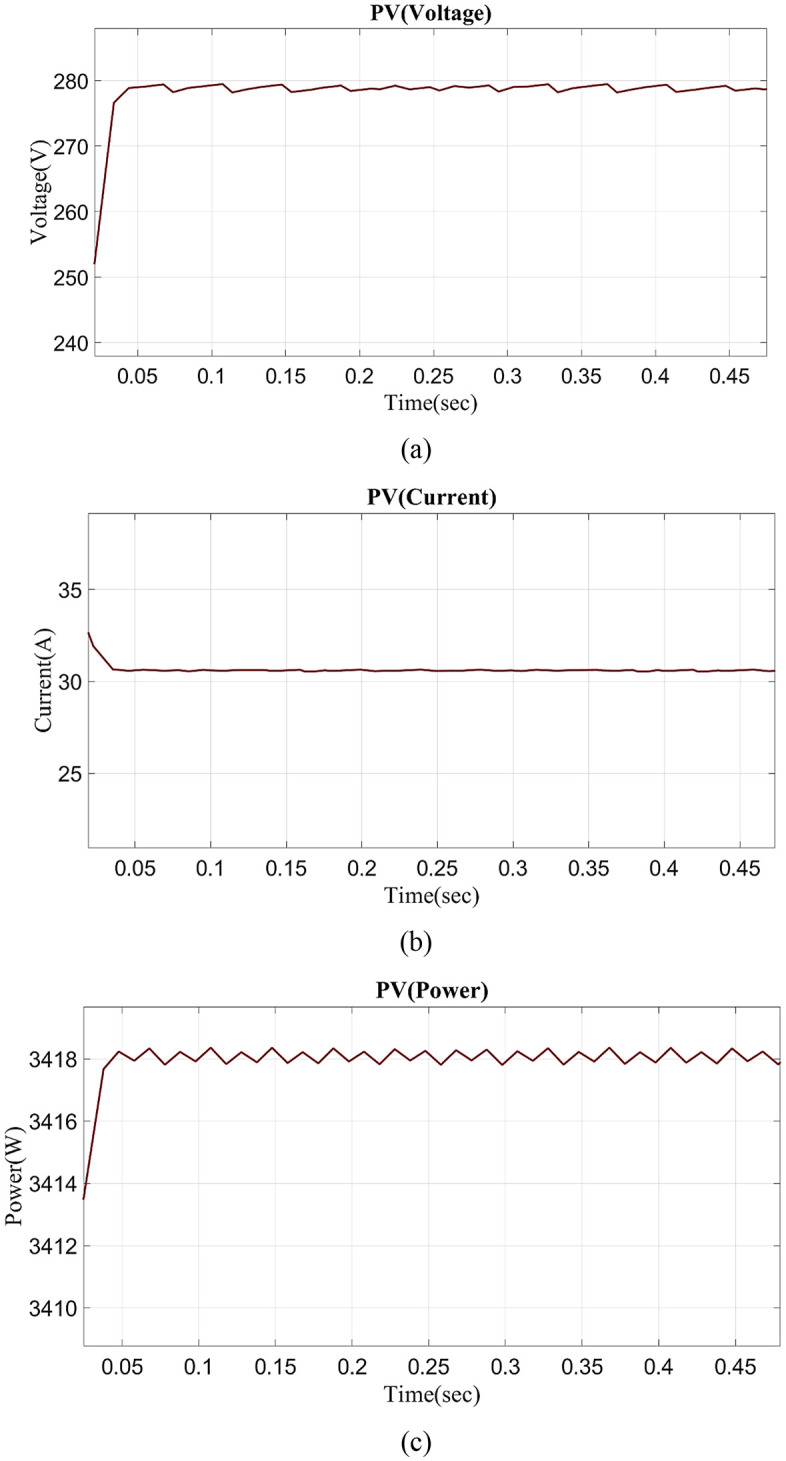
Evaluation of (a) PV current, (b) PV voltage, (c) PV power.

Fig 8(a) depicts the PV current vs time graph. PV current varies slightly from 0 to 0.03 seconds and 0.03 to 0.5 seconds while maintaining a constant 280V. Fig 8(b) depicts the PV voltage vs time graph. PV voltage varies from 0 to 0.03s and to constantly maintained in 0.03s to 0.5s at 30V. Fig 8(c) illustrates the PV power vs time graph. PV power fluctuates from 0 to 0.03s and steadily maintains 0.03s to 0.5s in 3480W.

**Fig 9:**
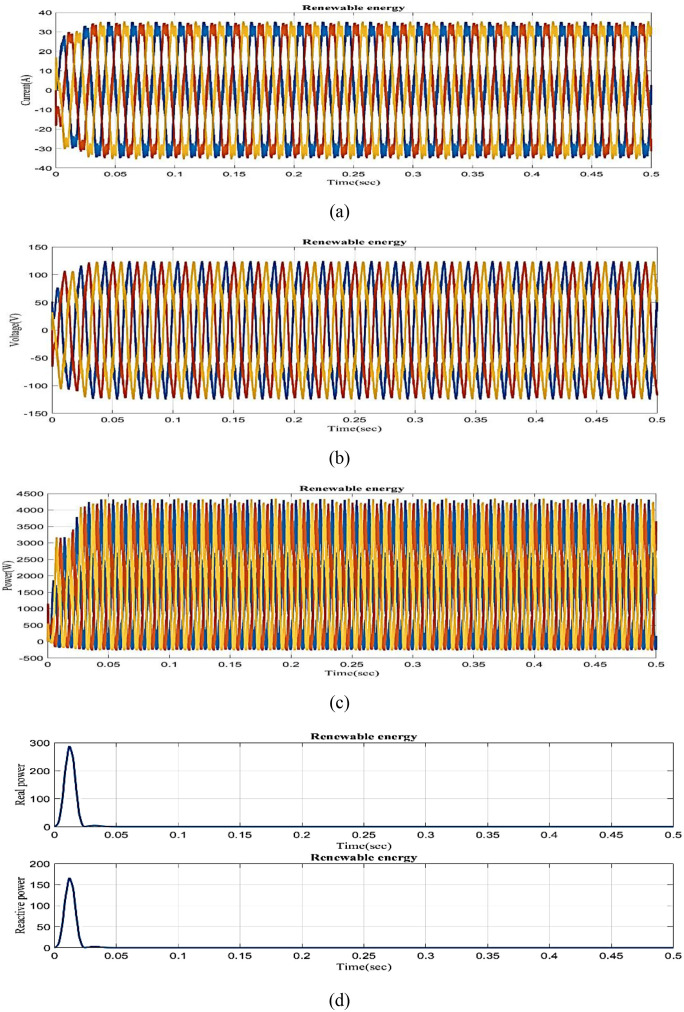
Analysis of (a) Renewable energy current, (b) Renewable energy voltage, (c) Renewable energy power, (d) real power and reactive power of RES.

The graph of renewable energy current versus time is shown in Fig 9(a). Renewable energy current fluctuates partially in 0–0.03 s and maintains a constant 35A current in 0.03–0.5 s. Fig 9(b) clearly shows the renewable energy voltage vs time graph. Renewable energy voltage fluctuates partially in 0–0.03 s and maintains a constant 120V in 0.03–0.5 s. The graph of renewable energy power vs time is shown in Fig 9(c). In 0 to 0.03s, power fluctuates partially, and from 0.03s to 0.5s, renewable energy power remains nearly maintained at 4300W. The graph of renewable energy vs real vs reactive power is clearly shown in Fig 9(d). In Fig 9(d), 0 to 0.03s real power reaches a peak of 290A and slightly decreases and maintains 0.1 W constantly in 0.03 to 0.5s. It shows that in 0 to 0.03s, reactive power peaks at 155 VAR, then steadily decreases to 0.1 VAR in 0.03 to 0.5s.

**Fig 10:**
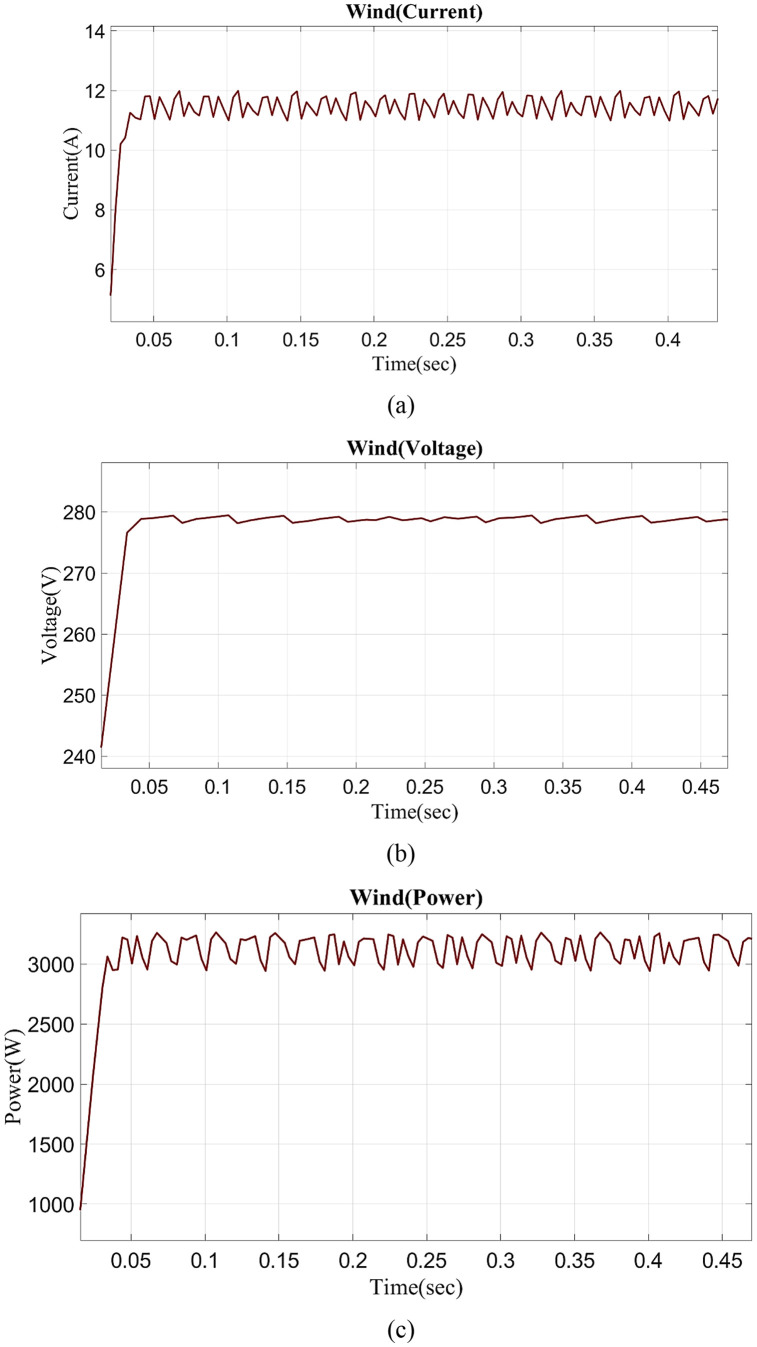
Evaluation of wind (a) current (b) voltage (c) power.

In Fig 10(a) clearly shows the wind current vs time graph. In 0 to 0.03s, the wind current slightly varies, and from 0.03s to 0.5s remains nearly at 12A. The wind voltage vs time graph is shown in Fig 10(b). Wind voltage fluctuates between 0–0.03s and is nearly constant at 280V in 0.03–0.5s. In Fig 10(c) shows the wind power vs time graph. In 0 to 0.03s, wind power slightly fluctuates, and from 0.03 to 0.5, sec nearly constant in 3100W.

A system with a current controller and a voltage controller to tackle current and voltage problems, respectively. Due to a problem with an unbalanced load in the grid, it is possible to use power quality with power factor, frequency, voltage deviation, and harmonics to modify the poor AC supply.

**Fig 11:**
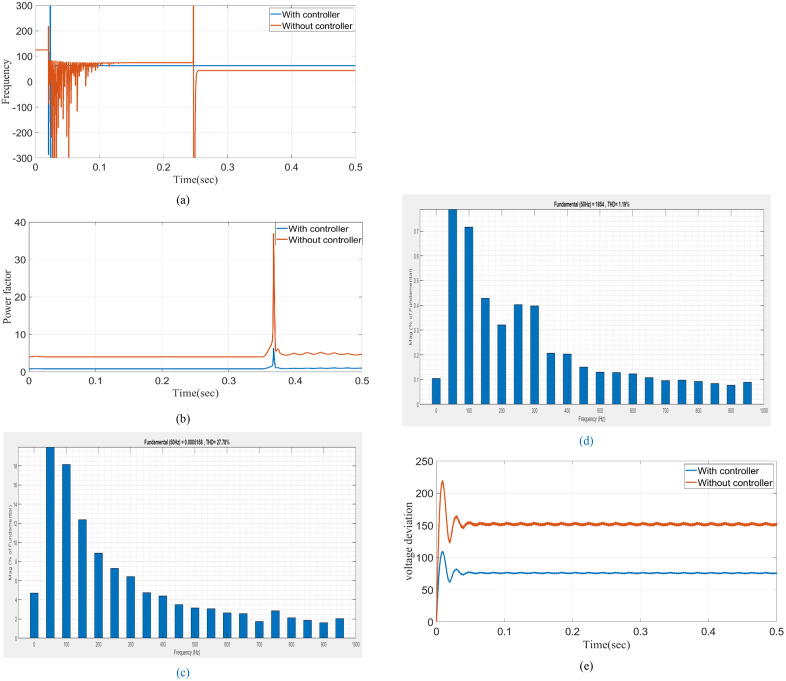
Analysis (a) frequency (b) power factor (c) THD without controller (d) THD with controller (e) voltage deviation.

The definition of power frequency fluctuations is the difference between the nominal value (50 or 60 Hz) of the power system’s fundamental frequency and that value. Frequency fluctuates in the absence of a controller. However, the proposed model efficiently keeps the frequency at 50 Hz within the controller, which is clearly shown in Fig 11(a). Power Factor (PF) is the proportion of working power (measured in kW) to apparent power (measured in kVA). With a controller, the power factor is consistently maintained at 0.8, but without a controller, there are minor swings shown in Fig 11(b). Integer multiples of the fundamental power frequency characterize harmonic currents or voltages. Fig 11(c) and 11(d) show that a proposed model that has a controller can operate well, whereas fluctuation still happens without a controller. Voltage deviation is the result of first anticipating the following signal value and then comparing the actual true value to the prediction. It shows that without the controller, the THD is 27%, and after the controller is fixed, the THD is 1.16%. Fig 11(e) demonstrates that a proposed model with a controller can function effectively, whereas fluctuation still occurs in the absence of a controller.

Table 3 displays the comparative study, and it is clear that the proposed controller has exhibited the least change in F, THD, VD, and PF concerning the PQ under grid-integrated circumstances. With the controller, 50 Hz, 0.8 p.f, 2.2% THD, and 70 V deviations are maintained.

**Table 3:** Comparison of with and without controller.

Parameter	Without controller	With controller
Frequency	60	50
Power factor	5	0.8
THD	3.6	2.2
Voltage deviation	160	70

#### 4.1.1. Sag condition.

**Fig 12:**
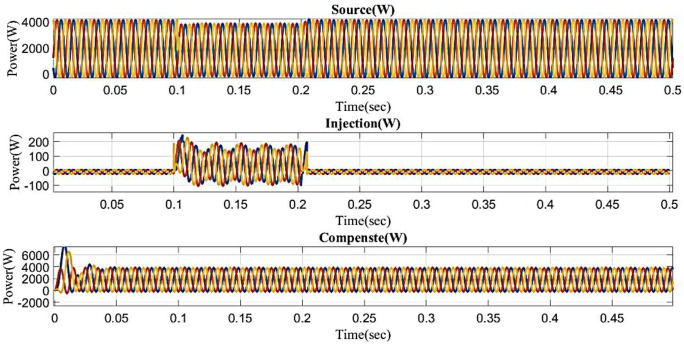
Performance in sag condition in simple microgrid structure.

The voltage drops below its steady state during the sag period, typically as a result of a systemic malfunction. During that time, power is injected to balance the load side, as shown in Fig 12. The generated voltage has sag concerns. In other words, although the supply voltage is flowing constantly and without interruption, there is a sag that arises every 0.1 to 0.2 seconds. It is based on an intelligent controller.

**Fig 13:**
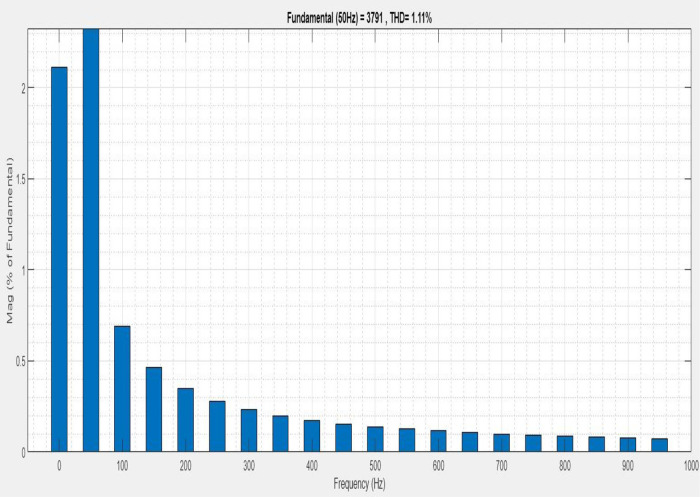
THD analysis with simple microgrid structure with sag condition.

Fig 13 illustrates an analysis of Total Harmonic Distortion (THD). Harmonics in a system can occur as a result of frequency changes. The proposed controller’s Simple microgrid model’s load current THD is 1.11%.

**Fig 14:**
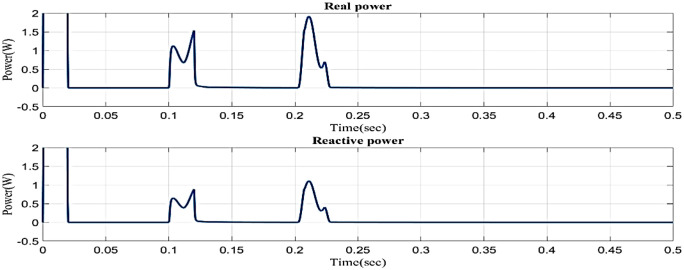
Real and reactive power in sag condition in simple microgrid structure.

Fig 14 illustrates the reactive power and real power concerning time in the sag condition. Real power varies in 0.1s to 0.13s and 0.2s to 0.23s and constantly maintains in 0.1 W in 0.23s to 0.5s. Reactive power varies in 0.1s to 0.13s and 0.2s to 0.23s, and 0.23s to 0.5s. Reactive power is constantly maintained in 0.1 VAR.

**Fig 15:**
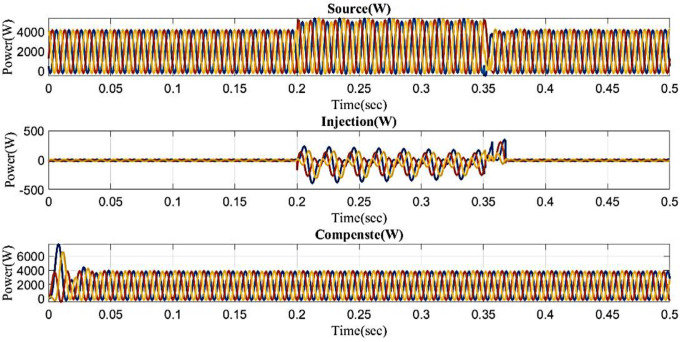
Performance in the swell condition in a simple microgrid structure.

Power that is increased above the level of constant voltage results in swell, a condition of poor power quality that significantly affects the load demand. The process of suggested controller-based simple microgrid design in swell conditions is shown in Fig 15. This illustrates that the swell occurs between 0.2and 0.36 seconds.

**Fig 16:**
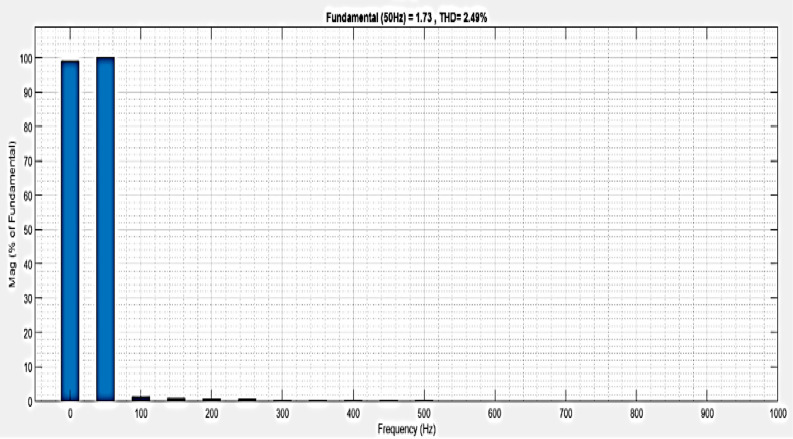
THD analysis with simple microgrid structure with swell condition.

Fig 16 illustrates the analysis of total harmonic distortion (THD) for a simple microgrid system. Changes in frequency can lead to harmonics in a system. The proposed controller based load current THD has been stated to be 1.36%.

**Fig 17:**
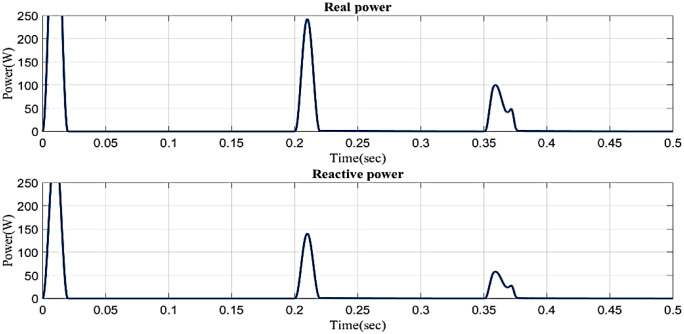
Real and reactive power in the swell condition in a simple microgrid structure.

In a sag state, Fig 17 shows the reactive power and real power with respect to time. Real power changes between 0 and 0.02 seconds, between 0.2 and 0.22 seconds, and between 0.35 and 0.38 seconds it remains constant at 0 A. Reactive power consistently maintains a steady level of 0A even though it fluctuates from 0 to 0.02 seconds, 0.2 to 0.22 seconds, and 0.35 to 0.38 seconds. It is based on an intelligent controller.

**Fig 18:**
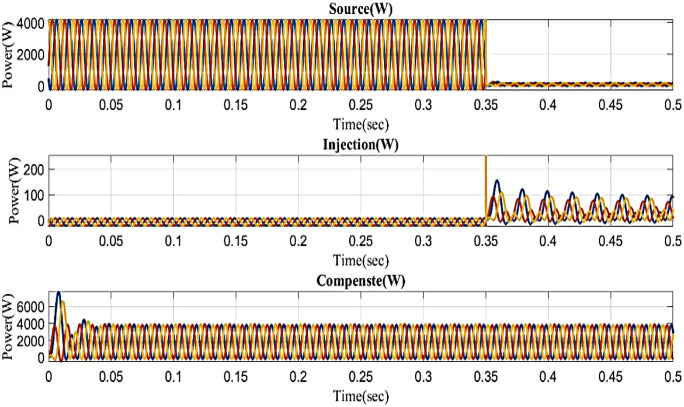
Performance in interruption condition in simple microgrid structure.

Because it also affects the components on the load side, interruption is also regarded as one of the system’s power quality issues. Fig 18 displays the interruption condition, the injected voltage, and the corrected load current. It demonstrates the interruption occurs to interrupt the power flow for 0.35 to 0.5 seconds based on the intelligent controller.

**Fig 19:**
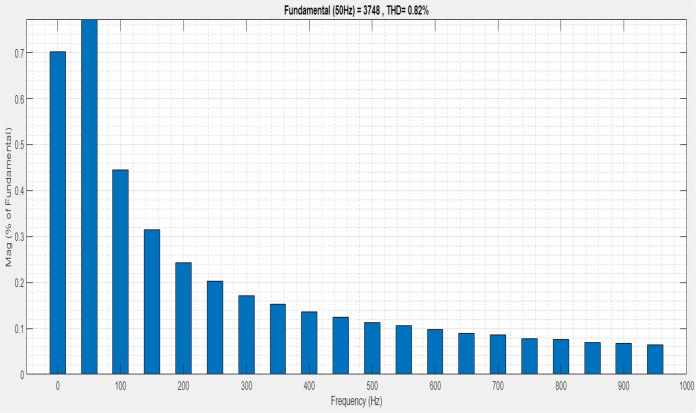
THD analysis with simple microgrid structure with interruption condition.

Analysis and demonstration of the interruption THD value are shown in Fig 19. In a simple microgrid, the structure reduces the harmonic content by 0.82% to protect the end user.

**Fig 20:**
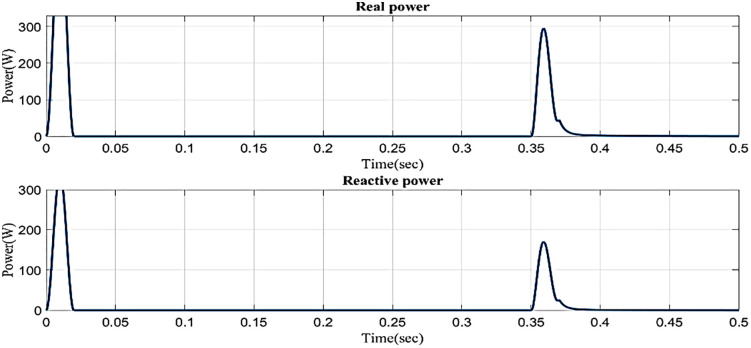
Real and reactive power in interruption condition in a simple microgrid structure.

In an interruption state, Fig 20 shows the reactive power and real power concerning time based on the proposed controller. Real power changes between 0 and 0.02 seconds, between 0.35 and 0.38 seconds, and between 0.38 and 0.5 seconds it remains constant at 0.1 W. Reactive power consistently maintains a steady level of 0.1VAR even though it fluctuates from 0 to 0.02 seconds and 0.35 to 0.38 seconds.

### 4.2. Microgrid with extra line impedances

For LV power systems, line resistance and reactance are fixed at 0.642 Ω/km and 0.083 Ω/km, respectively. The non-critical load (NCL), which is 6kW, is employed at 50 m line distance (0.0321 + j0.00415 Ω), and the critical load (CL), which is 4 kW, is located at 100 m line distance (0.0642 + j0.0083 Ω). By way of the station bus, DER2 and DER1 are substantially nearer to PCC. With a line length of 50 meters, CB4 connects the station bus to the main load bus, which has a 10 kW capacity. After installing the line impedance, PQ problems are introduced into the system to evaluate the performance of the suggested optimal controller. Each issue period, sag, swell, interruption, power factor, voltage deviation, THD, and frequency are analyzed.

**Fig 21:**
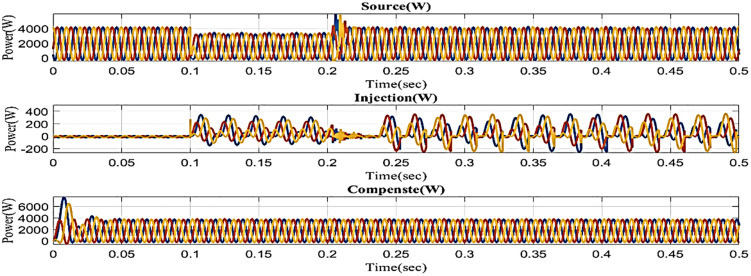
Sag condition performance in microgrid model with additional line impedance.

The voltage typically decreases below its constant level during the sag period as a result of a systemic failure. Because the generated voltage has sag issues, as seen in Fig 21, power is injected during that time to balance off the load demand. In other words, despite the supply voltage flowing continuously and without interruption, a sag arises every 0.1 to 0.2 seconds.

**Fig 22:**
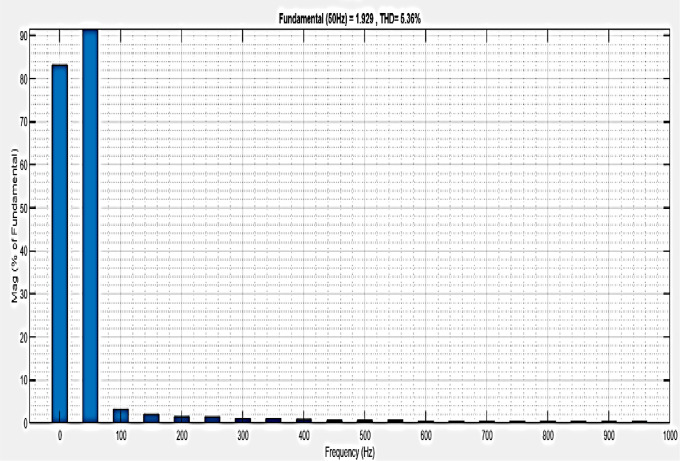
THD analysis with microgrid model with additional line impedance in sag condition.

Fig 22 shows a total harmonic distortion (THD) analysis. Frequency changes can cause harmonics in a system. The microgrid model with line impedance has a load current THD of 5.36%. It is based on an intelligent controller.

**Fig 23:**
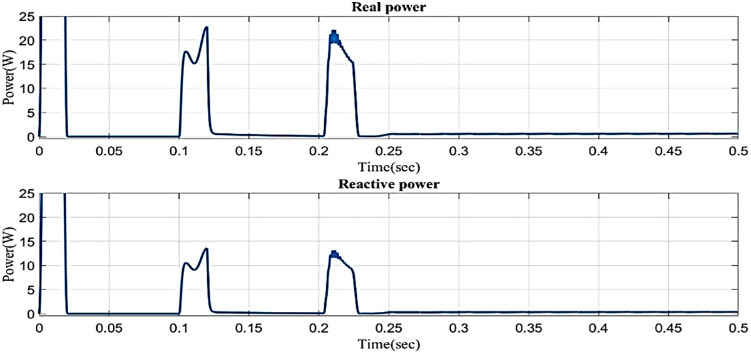
Real and reactive power in sag condition in microgrid model with line impedance.

Fig 23 illustrates the reactive and real power concerning time in sag condition in microgrid model with line impedance based on the proposed controller. Real power varies in 0.02s to 0.1s and 0.13s to 0.2s and is constantly maintained at 2 W in 0.23s to 0.5s. Reactive power varies in 0.1s to 0.13s and 0.2s to 0.23s and 0.23s to 0.5s. Reactive power is constantly maintained in 1VAR.

**Fig 24:**
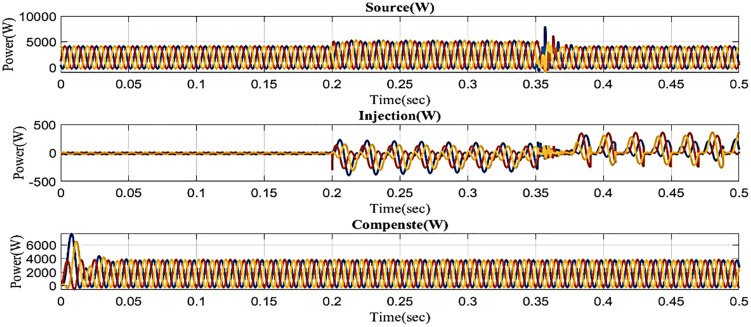
Swell condition performance in microgrid model with additional line impedance.

Swell is a low-quality power scenario where power is increased over the level of constant voltage, leading to serious issues at the load side. The performance of the microgrid with line impedance in swell conditions based proposed controller is shown in Fig 24. This demonstrates that the swell occurs between 0.2 and 0.35 seconds.

**Fig 25:**
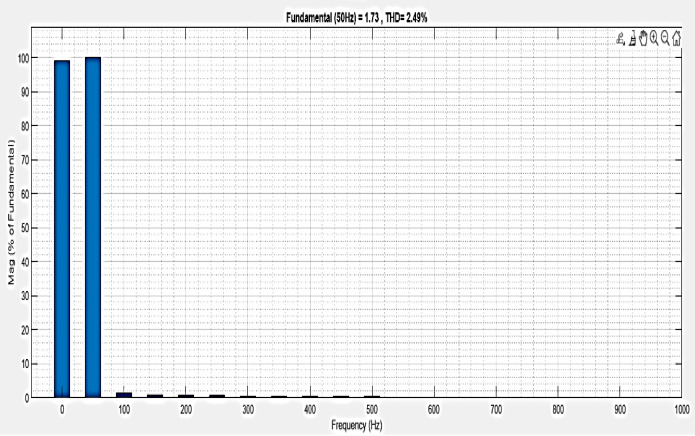
THD analysis with microgrid model with additional line impedance in swell condition.

The analysis of THD for a microgrid with a line impedance system is shown in Fig 25. Harmonics in a system can result from frequency changes. The load current THD is 2.49%, as stated.

**Fig 26:**
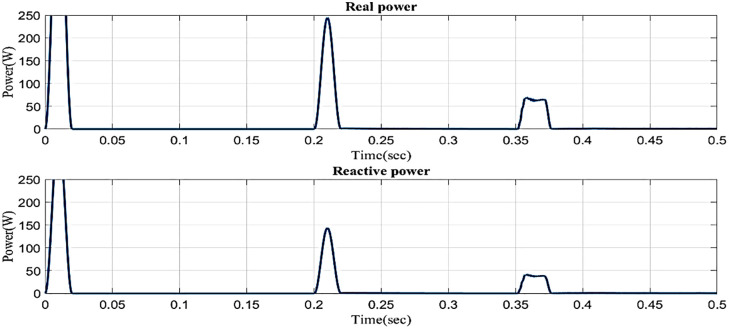
Real and reactive power in swell condition in microgrid model with line impedance.

Fig 26 illustrates the reactive and real power in the proposed controller-based microgrid model with line impedance during the swell scenario. Real power fluctuates between 0 and 0.02 seconds and 0.2 and 0.22 seconds, and 0.35 and 0.38 while remaining constant at 0.2 W between 0.38 and 0.5 seconds. Reactive power fluctuates between 0 and 0.02 seconds and 0.2 and 0.22 seconds, and 0.35 and 0.38 while remaining constant at 0.5 VAR between 0.38 and 0.5 seconds.

**Fig 27:**
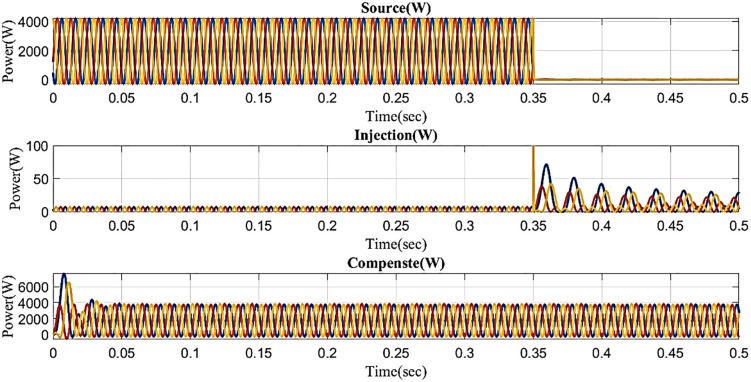
Performance in interruption condition in microgrid model with line impedance.

Because it also affects the components on the load side, interruption is also regarded as one of the system’s power quality issues. Fig 27 displays the interruption situation, the injected voltage, and the corrected load current. It demonstrates that the interruption occurs to interrupt the power flow for 0.35 to 0.5 seconds. Then, in order to compensate for the load current, the compensator injects power throughout the interruption by the output of the intelligent controller.

**Fig 28:**
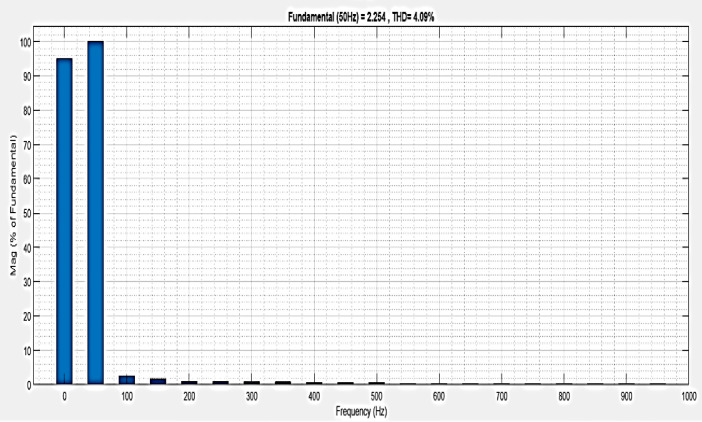
THD analysis with microgrid model with line impedance structure with interruption condition.

The intelligent controller based on total harmonic distortion (THD) is analyzed and shown in Fig 28. Frequency changes are the root cause of the system harmonics. The content of the harmonics changes along with the system frequency, which can lead to component damage and overheating. The proposed controller-based microgrid with line impedance that reduces voltage harmonics has a load current THD of 2.67%.

**Fig 29:**
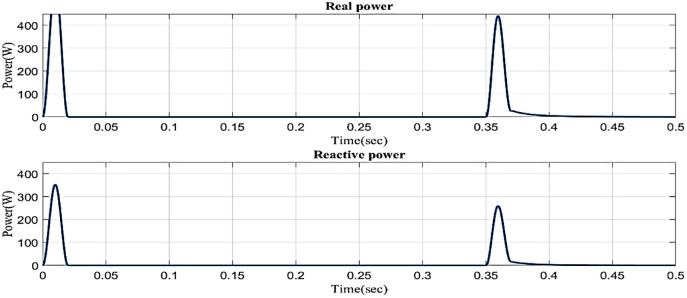
Real and reactive power in interruption condition in microgrid model with line impedance.

In an interruption state, Fig 29 shows the reactive power and real power concerning time based on the proposed controller. Real power changes between 0 and 0.02 seconds, between 0.35 and 0.38 seconds, and between 0.38 and 0.5 seconds it remains constant at 0.1 W. Reactive power consistently maintains a steady level of 0.9 VAR even though it fluctuates from 0 to 0.02 seconds and 0.35 to 0.38 seconds.

A device that can handle voltage and current issues separately. Due to a problem with an imbalanced load in the grid, it is feasible to employ power quality with frequency, voltage deviation, power factor and harmonics to modify the inadequate AC supply.

**Fig 30:**
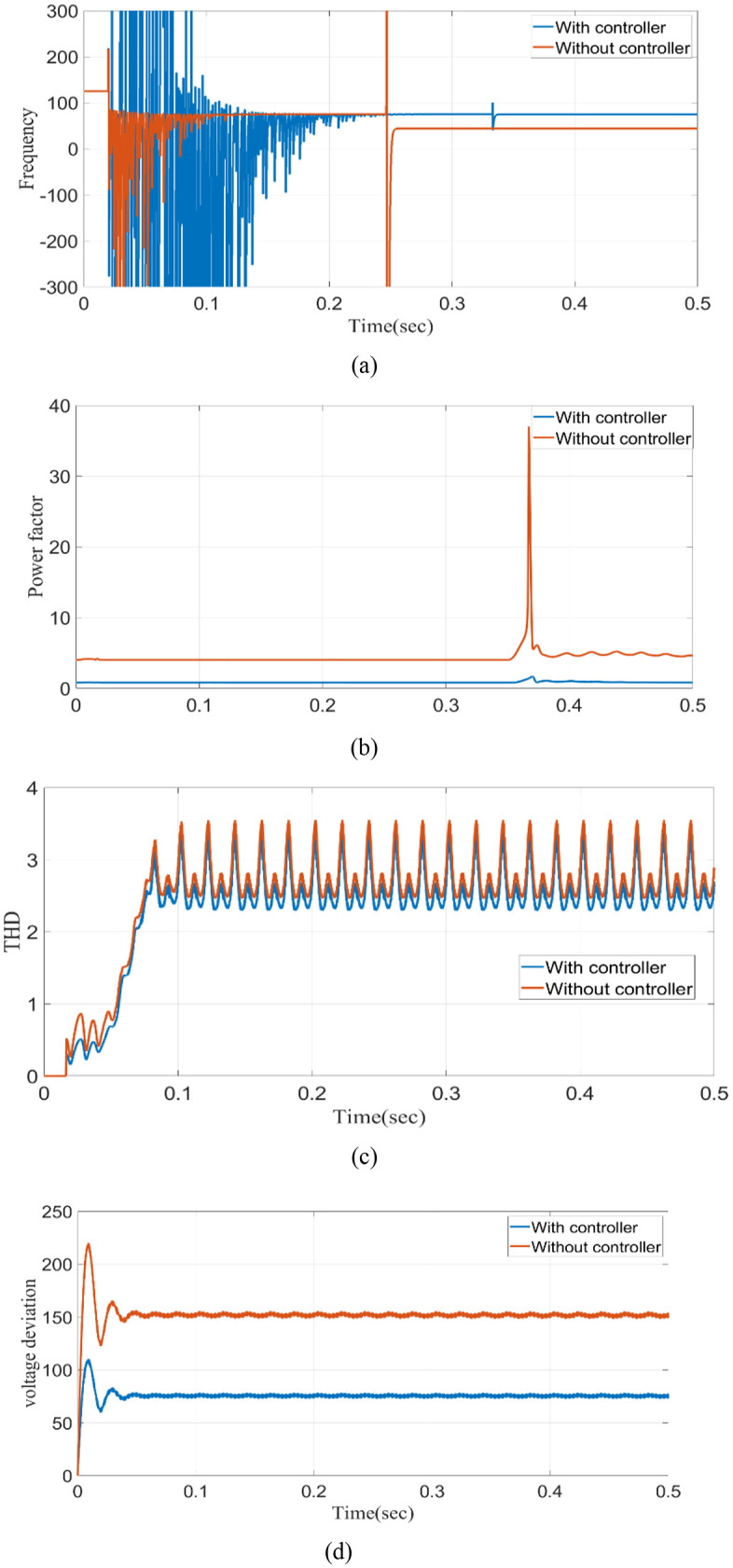
Analysis (a) frequency (b) power factor (c) THD (d) voltage deviation.

The proposed model effectively maintains the frequency at 50 Hz in the presence of a controller with the addition of line impedance in the starting period, as clearly illustrated in Fig 30(a). Still, frequency fluctuates in the absence of a controller. In addition to line impedance, the power factor is regularly maintained at 0.8 with a controller. Without a controller, however, there are slight swings, as seen in Fig 30(b). Fig 30(c) demonstrates that a proposed model addition of line impedance with a controller can function effectively in the starting period, whereas fluctuation still occurs in the absence of a controller. Fig 30(d) illustrates the effectiveness of the suggested model in addition of line impedance with a controller compared to the model without a controller, which still exhibits fluctuation. Comparison analysis of THD in the simple microgrid model and the microgrid model with line impedance is presented in Table 4 below.

**Table 4:** Comparison analysis of THD.

Condition	Simple microgrid model THD%	Microgrid model with line impedance THD%
Sag	1.11	6.36
Swell	2.49	2.49
Interruption	0.82	4.09

### 4.3. Realistic microgrid structure

A realistic microgrid structure, which includes genuine distribution network features like generating sources, loads on generating units, bus bars, line impedance, and the impact of converters and lines, can be used to verify the viability of the proposed concept. A multi-layer microgrid structure can be developed according to the enormous number of loads and DERs, enabling the microgrid to function at an operational point range. Ten sources with a utility grid is linked together to make a microgrid, and ten load impedances are linked in the system. Power sharing, source and wire losses, and the line impedance effect on PQ can all be investigated for any set of sites. It is possible to maintain a power balance among loads and sources to assess the Demand Response (DR) research. Simulation parameters is shown in Table 5. The proposed controller performance is validated under the grid connected and disconnected model.

**Table 5:** Simulation parameters in a realistic microgrid structure.

Operating area	Capacity	Load
MG1	4KW	load 1–2 KW
		load 2−1 KW
		load 3−1 KW
MG2	4KW	load 1–2 KW
		load 2−1 KW
		load 3−1 KW
MG3	6KW	load 1−1 KW
		load 2−2 KW
		load 3−1 KW

**Fig 31:**
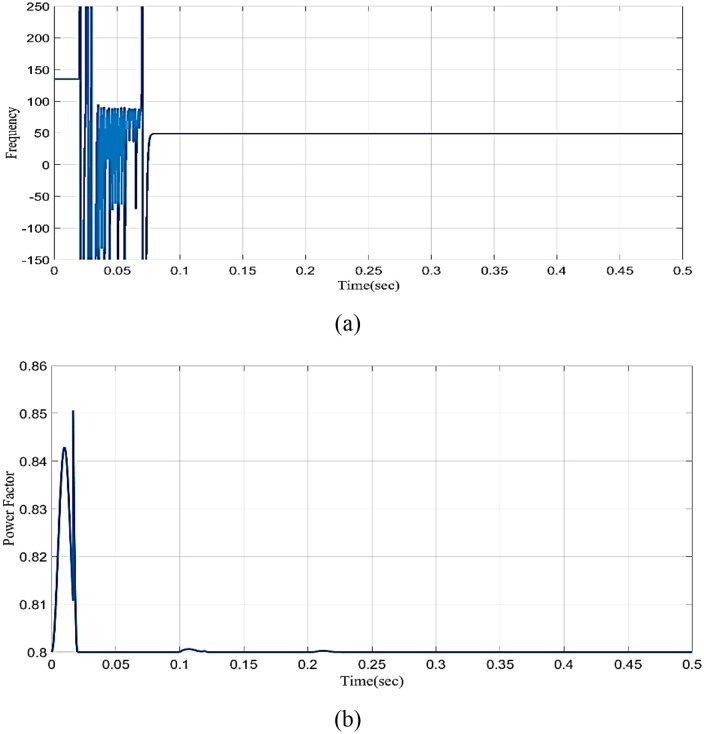
Analysis of (a) Frequency (b) Power factor in realistic microgrid structure.

Fig 31(a) shows the frequency graph in a realistic microgrid structure. In fault arise frequency varies concerning time in 0 to 0.07 seconds. The proposed controller effectively rectifies the fault. Fig 31(b) shows the power factor graph in a realistic microgrid structure. The power factor is the cosine of the angle between voltage and current. In fault frequency significantly varies from 0 to 0.02 seconds, and the proposed controller fixes the problem to maintain a power factor in the range of 0.8. This demonstrates that the proposed model provides a better outcome under all conditions.

Table 6 demonstrates the comparison of the proposed model with the state-of-the-art models for validating the process. The existing Phase Opposition Disposition (POD), Multiband inverter and Alternate Phase Opposition Disposition (APOD) [36] have voltage THD of 7.9%, 27% and 8.813% respectively. Its shows the proposed model have poor THD value than the traditional models.

**Table 6:** Comparison analysis of state-of-the-art methods THD.

Condition	Voltage THD%
Proposed FFO controller at Simple microgrid	1.11
Proposed FFO controller at the Microgrid model with line impedance	6.36
POD	7.9
APOD	8.813
Multiband	27

## 5. Conclusion

Power quality (PQ) issues in microgrids were addressed using a Fennec Fox Optimization (FFO)–based controller, tested under both grid-tied and off-grid modes. The controller regulates nine PQ parameters, including frequency, voltage variations, sag, swell, THD, power factor, and voltage stability. A microgrid model was developed with renewable sources, utility grid support, and a three-phase inverter. The FFO-based controller generates optimal PWM pulses using system voltage, current, switching frequency, and reference power. Simulation results show that the proposed system effectively manages PQ concerns. In a simple microgrid, THD was 1.11% (sag), 2.49% (swell), and 0.82% (interruption), while with line impedance, THD was 6.36%, 2.49%, and 4.09%, respectively. Communication delay and demand response were also considered for realistic operation. Although the method improves PQ, it suffers from design complexity and slower convergence. Future work will focus on AI-based robust controllers for faster and more precise performance.

## Nomenclature:

RES: Renewable Energy Sources
MGs: Micro Grids
PQ: Power Quality
THD: Total Harmonic Distortion
DER: Distributed Energy Resource
FFO: Fennec Fox Optimization
PI: Proportional Integral
PV: Photovoltaic
BESS: Battery Energy Storage System
PWM: Pulse Width Modulation
PLL: Phase Locked Loop
ANFIS: Adaptive Neuro Fuzzy Inference System
WES: Wind Energy System
SHF: Shunt Harmonic Filter
SG: Smart Grid
PSO: Particle Swarm Optimization
MPPT: Maximum Power Point Tracking
ADPM: Automatic Data Processing Machine
GSC: Grid Side Converter
PB: Power balance
IPM: Improved power management
GWO: Grey Wolf Optimization
UPQC: Unified Power Quality Conditioner
FOPID: Fractional Order Proportional Integral Derivative
FLC: Fuzzy Logic Controller
MPC: Model Predictive Controller
FCS; APQC: American Productivity and Quality Center;
TCSC: Thyristor Controlled Series Capacitor
SAPF: Shunt Active Power Filter
PCC: Point of Common Coupling
COA: Cheetah Optimized Algorithm
GZA: Gazelle Optimization Algorithm
APOD: Alternate Phase Opposition Disposition
